# A Novel Seventeen-Gene Metabolic Signature for Predicting Prognosis in Colon Cancer

**DOI:** 10.1155/2020/4845360

**Published:** 2020-11-17

**Authors:** Dakui Luo, Zezhi Shan, Qi Liu, Sanjun Cai, Qingguo Li, Xinxiang Li

**Affiliations:** ^1^Department of Colorectal Surgery, Fudan University Shanghai Cancer Center, Shanghai 200032, China; ^2^Department of Oncology, Shanghai Medical College, Fudan University, Shanghai 200032, China

## Abstract

A metabolic disorder is considered one of the hallmarks of cancer. Multiple differentially expressed metabolic genes have been identified in colon cancer (CC), and their biological functions and prognostic values have been well explored. The purpose of the present study was to establish a metabolic signature to optimize the prognostic prediction in CC. The related data were downloaded from The Cancer Genome Atlas (TCGA), Genotype-Tissue Expression (GTEx) database, and Gene Expression Omnibus (GEO) combined with GSE39582 set, GSE17538 set, GSE33113 set, and GSE37892 set. The differentially expressed metabolic genes were selected for univariate Cox regression and lasso Cox regression analysis using TCGA and GTEx datasets. Finally, a seventeen-gene metabolic signature was developed to divide patients into a high-risk group and a low-risk group. Patients in the high-risk group presented poorer prognosis compared to the low-risk group in both TCGA and GEO datasets. Moreover, gene set enrichment analyses demonstrated multiple significantly enriched metabolism-related pathways. To sum up, our study described a novel seventeen-gene metabolic signature for prognostic prediction of colon cancer.

## 1. Introduction

Colon cancer (CC) is the third most common cancer worldwide. Radical resection is considered the primary therapeutic strategy for the management of CC, followed by radiotherapy and chemotherapy. Yet, about 25%-40% of patients suffer recurrence after surgery and adjuvant chemotherapy and have a poor prognosis [1]. The tumor, lymph node, metastasis (TNM) staging system has been used as the standard classification for predicting the recurrence in patients with CC [2]. Nevertheless, this system is not ideal for the prognostic prediction and clinical management of CC. Thus, efforts have been made to develop new methods that could improve prognostic prediction and participate in making individualized decision using clinicopathologic characteristics and molecular biomarkers [3–5]. Among these new tools, gene score signatures based on integrated data analysis appear as a promising approach.

Metabolic reprogramming is considered one of the hallmarks of cancer [6]. Warburg effect, which is manifested as enhanced glucose uptake and lactate production, has been widely accepted as a common feature of metabolic reprogramming. Dysregulated expression of multiple rate-limiting enzymes may lead to activation of the Warburg effect in cancer [7–9]. Previous evidence indicated that altered metabolic genes or miRNAs modulate metabolic homeostasis in CC [10–12]. Moreover, abnormal changes in this signaling pathway direct a metabolic program of glycolysis in CC [13].

In the present study, the differentially expressed metabolic genes were selected using The Cancer Genome Atlas (TCGA) and Genotype-Tissue Expression (GTEx) databases. Then, a prognostic seventeen-metabolism related signature was developed from TCGA database and validated in the mix of GSE39582 set, GSE17538 set, GSE33113 set, and GSE37892 set by performing univariate Cox regression and lasso Cox regression analyses.

## 2. Materials and Methods

### 2.1. Microarray Data

Transcript data in TCGA-COAD were downloaded from https://portal.gdc.cancer.gov. Transcript data of normal colon tissues were obtained from the GTEx database, which contains normal tissue-specific gene expression. For the Gene Expression Omnibus (GEO) database, raw microarray cell intensity files of GSE39582, GSE17538, GSE33113, and GSE37892 were downloaded from the http://www.ncbi.nlm.nih.gov/geo/. The microarray data were background adjusted and normalized using Robust Multichip Average. All data were obtained from the same chip platform (Affymetrix Human Genome U133 Plus 2.0 Array). The EntrezGeneID was converted into the corresponding gene symbol according to the annotation platform. When multiple probes were matched to the same EntrezGeneID, the mean value was obtained on behalf of the average expression level. The ComBat method was applied to remove the potential batch effects. Clinical characteristics and prognostic information were collected.

### 2.2. Identification of Differentially Expressed Metabolic mRNA in TCGA-COAD and GTEx Database

The metabolism-related genes were retracted from KEGG gene sets that were associated with metabolism. Ultimately, 853 metabolism-related genes were overlapped in TCGA and GEO database. A total of 471 CC samples and 349 normal colon samples were selected and linear models for microarray data method was used to analyze differentially expressed metabolism-related genes (∣logFC | >0.5, *P* < 0.05).

### 2.3. Development of Risk Score for Metabolic Signature Using TCGA Database

Using the univariate Cox regression and lasso Cox regression model, a list of metabolic genes was identified and a multigene-based classifier for predicting prognosis was constructed. A formula of a risk score for each patient was presented with the expression levels of the genes and their corresponding coefficients. Patients were divided into high-risk and low-risk groups using the median value of risk score as the cutoff point.

### 2.4. External Validation of Risk Score Using the GEO Database

GSE39582, GSE17538, GSE33113, and GSE37892 datasets were combined for external validation. The risk score of included patients was calculated based on a formula derived from TCGA database. Patients were divided into high-risk and low-risk groups by using the median value of the risk score of the GEO database as the cutoff point.

### 2.5. Gene Set Enrichment Analyses

Gene Set Enrichment Analyses (GSEA) were performed to explore potential pathways related to the gene signature using the TCGA-COAD cohort. A *P* < 0.05 and FDR *q* < 0.25 were considered significant difference.

### 2.6. Statistical Analysis

Survival differences were evaluated using the Kaplan–Meier estimate with the log-rank test. Multivariate Cox regression analysis was performed to identify independent prognostic factors. The receiver operating characteristic (ROC) curve was plotted to investigate the prognostic or predictive accuracy of this signature. All statistical analyses were performed with R (version 3.6.1, https://www.r-project.org/).

## 3. Results

### 3.1. Development of Prognostic Risk Score for Metabolic Signature

**Figure 1:**
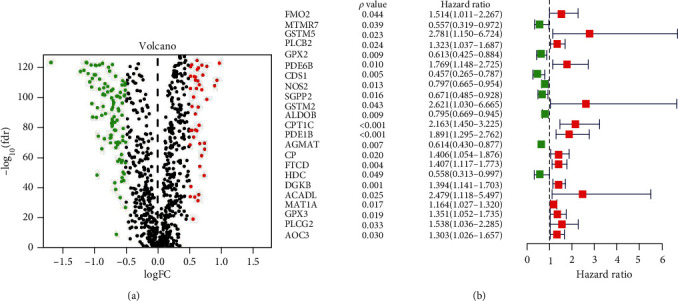
(a) Differentially expressed metabolism-related genes between colon cancer and normal tissues from TCGA and GTEx datasets. (b) The univariate Cox regression model revealed metabolism-related genes, which were related to prognosis.

A total of 471 CC samples and 349 normal colon samples, including 853 metabolism-related genes were included in the final analysis. Transcriptome change profiling was performed between CC samples and normal colon samples. A total of 147 genes (43 upregulated mRNAs and 104 downregulated mRNAs) were differentially expressed between the two groups (Figure 1(a)). Univariate Cox regression model and lasso Cox regression model identified 17 genes, which were used to construct the prognostic model (Figure 1(b)). The risk score was as follows: riskscore = (−0.221757031158657 × expressionlevelofMTMR7) + (0.206248657005617 × expressionlevelofGSTM5) + (−0.417679119748783 × expressionlevelofGPX2) + (0.150215117279366 × expressionlevelofPDE6B) + (−0.070604304617128 × expressionlevelofCDS1) + (−0.351801273605734 × expressionlevelofSGPP2) + (0.568971606841924 × expressionlevelofGSTM2) + (−0.111818090438981 × expressionlevelofALDOB) + (0.123891815430814 × expressionlevelofCPT1C) + (0.26051604643361 × expressionlevelofPDE1B) + (−0.0955344571364628 × expressionlevelofAGMAT) + (0.3138054768187 × expressionlevelofFTCD) + (−0.630585032132511 × expressionlevelofHDC) + (0.190772635324619 × expressionlevelofDGKB) + (0.326618039980251 × expressionlevelofACADL) + (0.130179173907145 × expressionlevelofMAT1A) + (0.251928635630125 × expressionlevelofPLCG2).

### 3.2. The Prognostic Value of Seventeen-Gene Metabolic Signature in Training and External Validation Series

**Figure 2:**
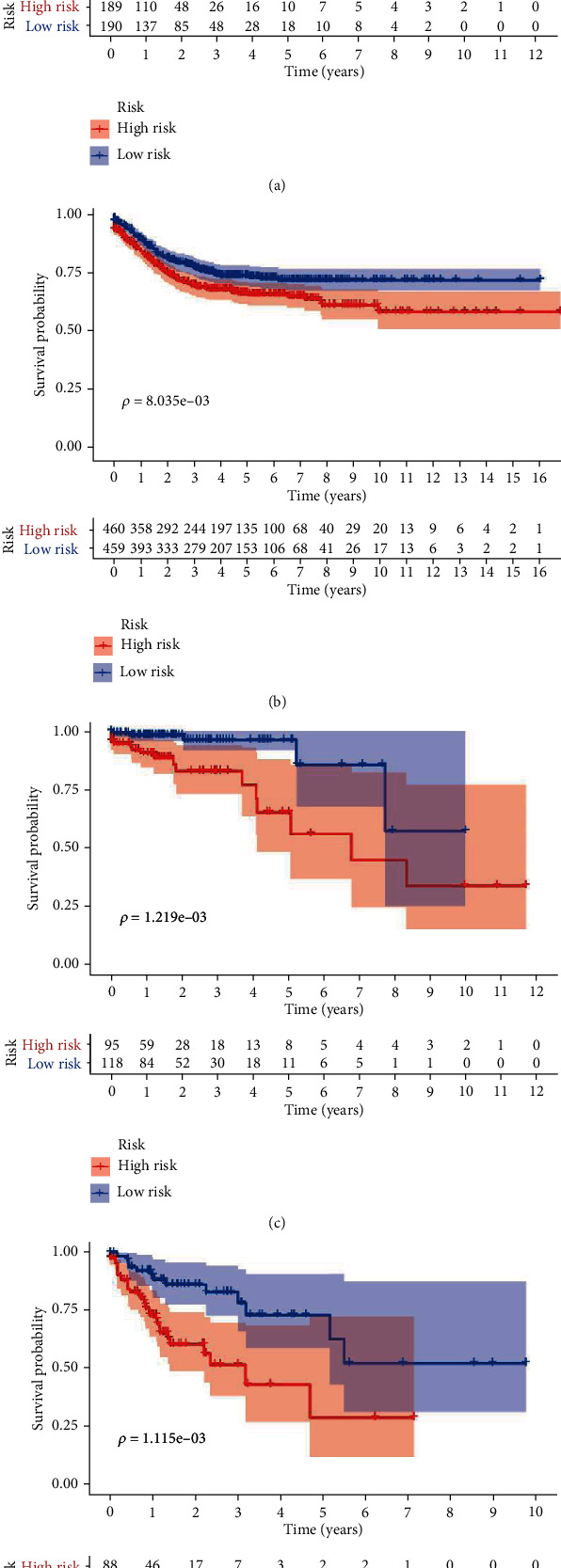
Kaplan–Meier estimates of colon cancer patients using the metabolic signature: (a) TCGA database; (b) incorporative GEO database; (c) stage I, II in TCGA database; (d) stage III, IV in TCGA database.

Patients in TCGA set were divided into the low-risk group (*N* = 190) and the high-risk group (*N* = 189) using the median risk score as the cutoff value. Patients with lower risk scores generally had a better prognosis than those with higher risk scores (hazard ratio (HR): 3.358, 95% confidence interval (CI): 2.506-4.501, *P* < 0.001, Figure 2(a)). To evaluate the universality of the metabolic signature, GEO datasets were used to validate the prognostic value. GEO datasets were combined with the GSE39582 set, GSE17538 set, GSE33113 set, and GSE37892 set for further analysis. As expected, patients in the high-risk group showed poorer prognosis compared to those in the low-risk group (HR: 1.174, 95% CI: 1.093-1.262, *P* < 0.001) (Figure 2(b)). In TCGA set, stratified analysis demonstrated that the metabolic classifier still presented significant prognostic difference in stage I, II and stage III, IV (Figures 2(c) and 2(d)).

**Figure 3:**
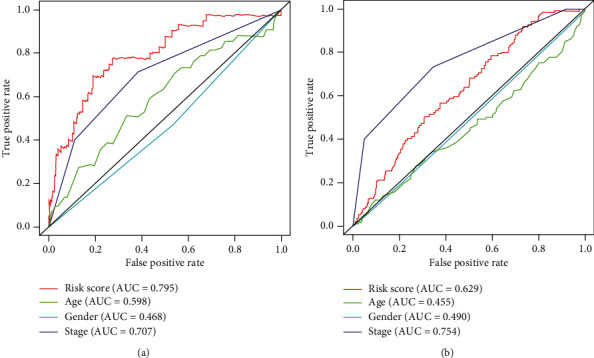
Receiver operating characteristic (ROC) analysis of the sensitivity and specificity of the metabolic signature and clinicopathological features: (a) TCGA database; (b) incorporative GEO database.

**Figure 4:**
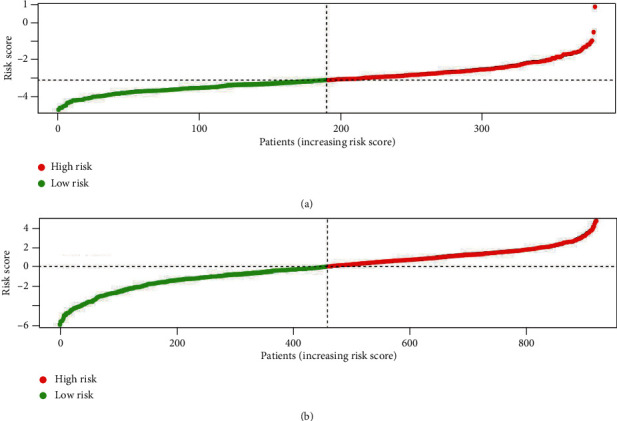
The metabolic signature risk score distribution: (a) TCGA database; (b) incorporative GEO database.

**Figure 5:**
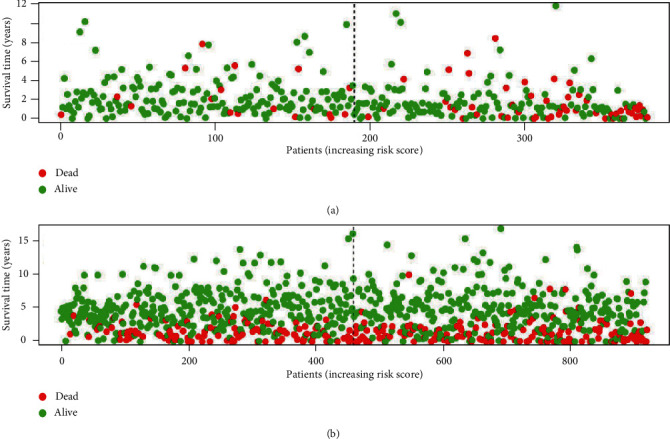
The distribution of patients' survival status and time: (a) TCGA database; (b) incorporative GEO database.

**Figure 6:**
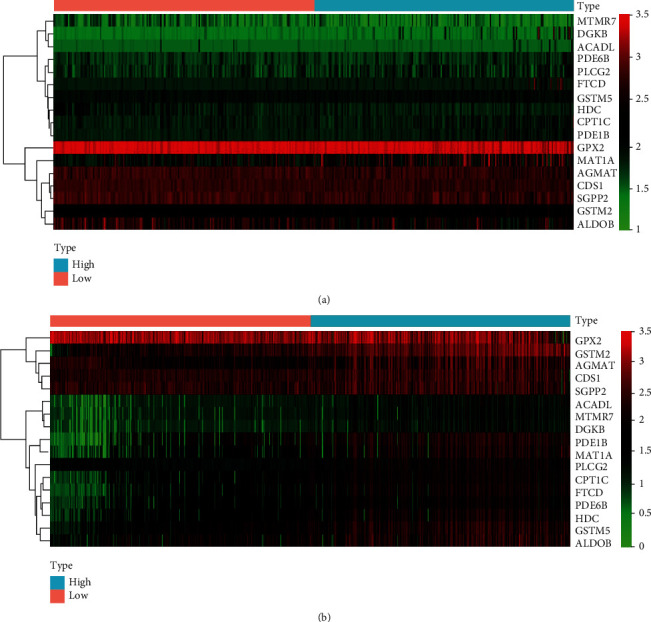
Heat map of the metabolic gene expression profiles: (a) TCGA database; (b) incorporative GEO database.

Furthermore, ROC analysis was done to assess the prognostic accuracy of the metabolic signature; briefly, the metabolic signature (area under curve (AUC) = 0.795) had better prognostic accuracy than the AJCC TNM stage in TCGA set (AUC = 0.707) (Figure 3(a)). Moreover, ROC analysis was performed to evaluate the prognostic accuracy of the metabolic signature (Figure 3(b)). The distribution of the gene risk score, the survival status of the colon cancer patients, and gene expression differences between the two groups were also shown (Figures 4–6).

### 3.3. Independent Prognostic Value of the Metabolic Signature

In TCGA dataset, univariate and multivariate Cox regression analyses demonstrated that TNM stage and the metabolic signature were independent prognostic factors for the overall survival. In the combined GEO database, identical results were observed (Table 1).

### 3.4. Identification of Signaling Pathways Related to the Metabolic Signature Using GSEA

**Figure 7:**
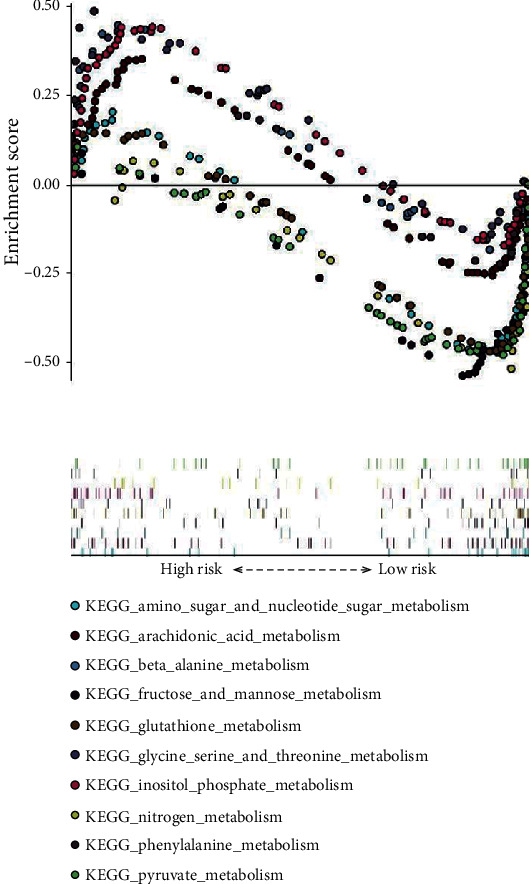
Ten representative-enriched KEGG pathways in TCGA database by GSEA.

GSEA were performed using TCGA dataset, and 38 enriched pathways were found to be related to the metabolic signature (Figure 7). A number of the enriched pathways were metabolism related, such as gluconeogenesis, fructose and mannose metabolism, nitrogen metabolism, and glycosaminoglycan biosynthesis. Moreover, several common pathways were also enriched, such as basal cell carcinoma, hedgehog signaling pathway, TGF-beta signaling pathway, and Wnt signaling pathway. Particularly, the majority of nonmetabolism-related pathways were enriched in the high-risk group, while the main metabolism-related pathways were enriched in the low-risk group.

### 3.5. Development of a Nomogram for Predicting Prognosis in Colon Cancer

**Figure 8:**
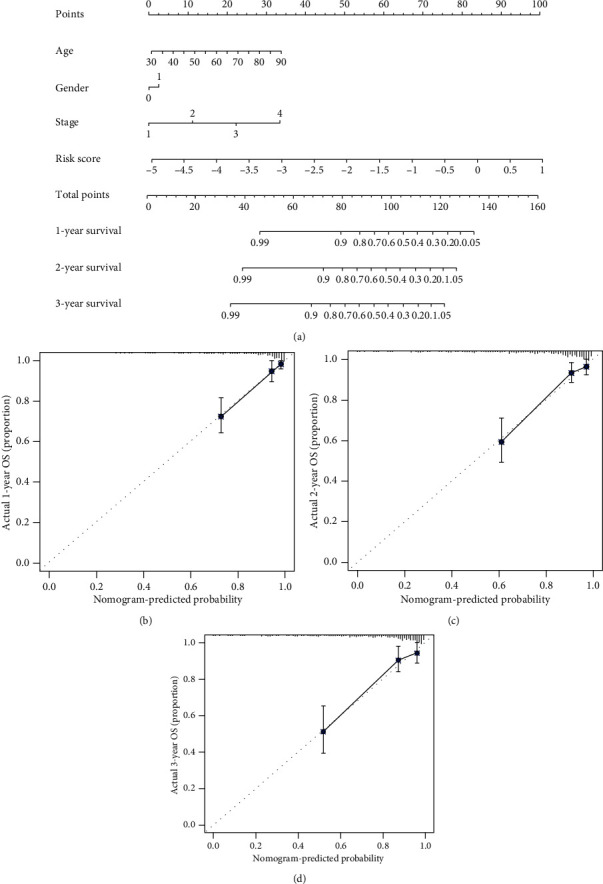
(a) Nomogram predicting prognosis of colon cancer patients from TCGA database. (b) The calibration plot of the nomogram (1 year). (c) The calibration plot of the nomogram (2 years). (d) The calibration plot of the nomogram (3 years).

To optimize prognostic prediction, a nomogram which integrated the metabolic signature, stage, age, and gender was developed based on TCGA dataset. The total points which were figured out using each variable could be converted to predict 1-, 2-, and 3-year probability of overall survival of colon cancer patients (Figure 8).

## 4. Discussion

Colon cancer is characterized by genetic heterogeneity and identical pathological stage with distinctive outcomes. So far, efforts have been made to explore sensitive biomarkers for eliminating deficiency of clinicopathological characteristics and optimizing the prognostic prediction of cancer. Recently, mRNA, miRNA, lncRNA, circRNA, and integrated signatures have been developed for prognostic prediction of CC [14–16]. Gene signatures in relation to specific phenotypes, such as immune infiltration, metastasis, and autophagy [17–19], have received increasing attention. Previous studies have demonstrated that metabolic disorders may contribute to tumor progression. In our present study, we developed a novel metabolic signature and validated its prognostic value in CC patients using TCGA and GEO datasets.

Based on the metabolic signature, patients were divided into the high-risk group and the low-risk group. Patients in the high-risk group presented poorer prognosis. ROC analysis suggested that the metabolic signature had better prognostic accuracy than the AJCC TNM stage. What is more, the metabolic signature was an independent prognostic factor for CC patients in both training sets and validation set, thus could be used as a promising biomarker for optimizing prognostic prediction of CC. The GSEA results revealed that many enriched pathways were associated with the metabolic signature. The majority of enriched pathways were metabolism related, indicating that the signature was a marker of the dysregulated metabolic microenvironment of CC. Intriguingly, the metabolism-related pathways were enriched in the low-risk group. These data suggest that targeted metabolic therapy may be more effective for patients in the low-risk group, while patients in the low-risk group may benefit from nonmetabolic intervention. Nevertheless, the underlying molecular mechanisms still need to be explored.

Most of the genes of our metabolic signature have been reported to be associated with cancer. Myotubularin-related protein 7 (MTMR7) is downregulated in colorectal cancer and is a negative predictor of colorectal cancer patient survival. Mechanically, MTMR7 inhibits insulin-mediated AKT-ERK1/2 signaling, which in turn decreases colorectal cancer cell proliferation [20].

Glutathione peroxidase 2 (GPX2), which belongs to the antioxidant enzyme glutathione peroxidase family, is often upregulated in multiple tumors [21–24]. In colon cancer, high GPX2 expression has been associated with early tumor recurrence, and H_2_O_2_ neutralization by GPX2 has been identified as essential for maintaining clonogenic and metastatic capacity [25].

CDS1 is an enzyme that is essential for the regeneration of the signaling molecule PIP2 from phosphatidic acid. Most of the CpG sites in the promoter area of the CDS1 gene are methylated in the majority of the cancer tissues. Moreover, decreased expression of CDS1 was detected in hepatocellular carcinoma [26].

Glutathione S-transferase mu2 (GSTM2) is a phase II detoxification enzyme, which is often downregulated in lung cancer due to hypermethylation of its promoter. GSTM2 has shown the ability to increase the expression of CCN2 and inhibit lung cancer migration [27].

Previous evidence has indicated that ALDOB has a dual function in tumor progression. On the one hand, ALDOB inhibits metastasis through TET1 in hepatocellular carcinoma [28]; on the other hand, it enhances fructose metabolism and drives metabolic reprogramming of colon cancer liver metastasis [29]. Interestingly, both univariate and multivariate regression analyses in our previous study revealed that high ALDOB expression was associated with poor overall survival and disease-free survival of colorectal cancer patients. Further experiments demonstrated that the upregulation of ALDOB promotes tumor progression by epithelial-mesenchymal transition in colorectal cancer [30]. In line with our previous study, we verified a significant upregulation of ALDOB in colon cancer using TCGA and GTEx datasets.

Carnitine palmitoyltransferase 1C (CPT1C), an enzyme that has an important role in the beta-oxidation of long-chain fatty acids, has been shown to regulate cancer cell senescence through mitochondria-associated metabolic reprogramming [31]. Moreover, CPT1C promotes cancer cell growth and metastasis in papillary thyroid carcinomas under conditions of metabolic stress [32].

Agmatinase (AGMAT) functions as an intermediary in polyamine biosynthesis. A recent study suggested that AGMAT could promote lung cancer progression by activating the NO-MAPKs-PI3K/Akt pathway [33]. In colon cancer, AGMAT promotes tumor progression by inducing chronic inflammation [34].

Formiminotransferase cyclodeaminase (FTCD) is an enzyme, which comprises two domains (FT and CD) and catalyzes histidine degradation during folate metabolism [35]. The enzyme is most highly expressed in the liver. FTCD was identified as a new regulator for HIF-1*α* in a hepatocarcinoma cell line [36].

L-histidine decarboxylase (HDC) is the rate-limiting enzyme for the generation of histamine by decarboxylating L-histidine [37]. Previous studies revealed that polymorphisms of the HDC gene were significantly associated with breast cancer in the Chinese Han population, thus enhancing its role as a promising diagnostic or therapeutic target for breast cancer [38].

ACADL is in charge of lipid and energy metabolism [39]. Abnormally expressed ACADL has been detected in prostate cancer and esophageal squamous cell carcinoma [40, 41]. Nevertheless, its regulatory mechanism in malignant diseases remains unclear.

Methionine adenosyltransferase 1a (MAT1A) is an enzyme that has a vital role in the methylation cycle by regulating S-adenosylmethionine [42]. MAT1A, which can enhance cell survival under chemotherapy, has been associated with drug resistance in bladder cancer PDX mice [43]. Torres et al. found that dysregulated MAT1A gene expression resulted in a specific pattern of promoter methylation and histone acetylation [44]. Yet, data on the role of GSTM5, PDE6B, SGPP2, PDE1B, DGKB, and PLCG2 in cancer are still lacking. Thus, further studies are needed to explore their molecular mechanisms in cancer.

This study has a few limitations. Firstly, the prognostic indicators are overall survival (OS) and relapse-free survival (RFS) in TCGA and GEO datasets, respectively. There are some differences between OS and RFS. Secondly, several critical prognostic factors, including differentiation and positive lymph node ratio, were unavailable in the public datasets, which may affect the multivariate Cox regression analysis. Thirdly, the model based on the 17 genes performed better on the training set, while much worse on the validation set. The data from TCGA were totally different from microarray. This difference was further displayed by serious imbalance of risk score between TCGA and GEO datasets. External validation of our metabolic signature is needed in more independent cohorts. Lastly, the functional experiments are needed to clarify the underlying molecular mechanism of our metabolic signature.

## 5. Conclusions

In conclusion, our study established a novel metabolic signature for optimization of prognostic prediction in colon cancer, which may contribute to clinical decision-making and generation of individual strategies.

## Figures and Tables

**Table 1:** Univariate and multivariable Cox regression analyses in colon cancer.

Variable	Univariate analysis		Multivariate analysis	
	HR (95% CI)	*P*	HR (95% CI)	*P*
Training set				
Metabolic signature	3.358 (2.506-4.501)	<0.001	3.253 (2.366-4.473)	<0.001
Age	1.029 (1.007-1.053)	0.011	1.040 (1.017-1.063)	<0.001
Gender	1.276 (0.780-2.089)	0.332	1.192 (0.723-1.966)	0.490
Stage	2.176 (1.645-2.877)	<0.001	2.202 (1.630-2.973)	<0.001
External validation set				
Metabolic signature	1.174 (1.039-1.262)	<0.001	1.098 (1.015-1.189)	0.020
Age	0.992 (0.983-1.001)	0.070	1.001 (0.992-1.010)	0.832
Gender	1.132 (0.885-1.448)	0.322	1.262 (0.983-1.619)	0.067
Stage	2.871 (2.428-3.396)	<0.001	2.786 (2.341-3.315)	<0.001

## Data Availability

The datasets included in our current study are available in Genotype-tissue expression (GTEx) (https://www.gtexportal.org/home/index.html), TCGA-COAD (https://portal.gdc.cancer.gov), GSE39582 (https://www.ncbi.nlm.nih.gov/geo/query/acc.cgi?acc=GSE39582), GSE17538 (https://www.ncbi.nlm.nih.gov/geo/query/acc.cgi?acc=GSE17538), GSE33113 (https://www.ncbi.nlm.nih.gov/geo/query/acc.cgi?acc=GSE33113), and GSE37892 (https://www.ncbi.nlm.nih.gov/geo/query/acc.cgi?acc=GSE37892).
